# Galactose and its Metabolites Deteriorate Metaphase II Mouse Oocyte Quality and Subsequent Embryo Development by Disrupting the Spindle Structure

**DOI:** 10.1038/s41598-017-00159-y

**Published:** 2017-03-22

**Authors:** Mili Thakur, Faten Shaeib, Sana N. Khan, Hamid-Reza Kohan-Ghadr, Roohi Jeelani, Sarah R. Aldhaheri, Bernard Gonik, Husam M. Abu-Soud

**Affiliations:** 1 Department of Obstetrics and Gynecology, The C.S. Mott Center for Human Growth and Development, Wayne State University School of Medicine, Detroit, MI 48201 USA; 2 Division of Genetic and Metabolic Disorders, Department of Pediatrics and Center for Molecular Medicine and Genetics, Wayne State University School of Medicine, Detroit, MI 48201 USA; 3 Department of Biochemistry and Molecular Biology, Wayne State University School of Medicine, Detroit, MI 48201 USA

## Abstract

Premature ovarian insufficiency (POI) is a frequent long-term complication of classic galactosemia. The majority of women with this disorder develop POI, however rare spontaneous pregnancies have been reported. Here, we evaluate the effect of D-galactose and its metabolites, galactitol and galactose 1-phosphate, on oocyte quality as well as embryo development to elucidate the mechanism through which these compounds mediate oocyte deterioration. Metaphase II mouse oocytes (n = 240), with and without cumulus cells (CCs), were exposed for 4 hours to D-galactose (2 μM), galactitol (11 μM) and galactose 1-phosphate (0.1 mM), (corresponding to plasma concentrations in patients on galactose-restricted diet) and compared to controls. The treated oocytes showed decreased quality as a function of significant enhancement in production of reactive oxygen species (ROS) when compared to controls. The presence of CCs offered no protection, as elevated ROS was accompanied by increased apoptosis of CCs. Our results suggested that D-galactose and its metabolites disturbed the spindle structure and chromosomal alignment, which was associated with significant decline in oocyte cleavage and blastocyst development after *in-vitro* fertilization. The results provide insight into prevention and treatment strategies that may be used to extend the window of fertility in these patients.

## Introduction

**Figure 1:**
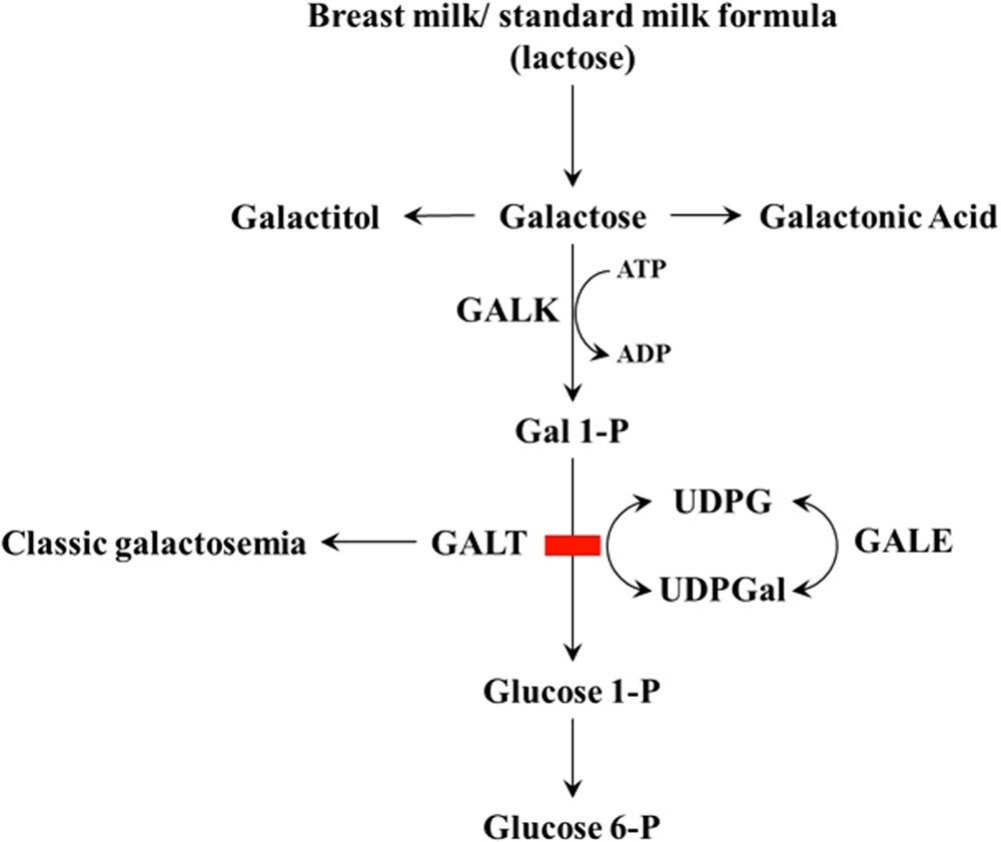
The Leloir pathway of galactose metabolism. GALT, Galactose 1-phosphate uridyltransferase; GALE, UDP-galactose 4′-epimerase; GALK, Galactokinase; UDPGal, UDP-galactose; UDPG, UDP-glucose; Gal-1-P, Galactose 1-phosphate; Glucose-1-P, Glucose-1-phosphate; Glucose-6-P, Glucose-6-phosphate.

Classic galactosemia is an inborn error of metabolism caused by deficiency of the enzyme galactose 1-phosphate uridyl transferase (GALT) in the Leloir pathway of galactose metabolism (Fig. 1), which includes three enzymes: galactokinase (GALK), galactose 1-phosphate uridyltransferase (GALT) and UDP-galactose 4-epimerase (GALE). Deficiency of GALT results in accumulation of galactose and its metabolites, galactose 1-phosphate and galactitol (generated when galactose is reduced by aldose reductase).

A majority of women with this disorder develop premature ovarian insufficiency (POI) despite adequate dietary restrictions^1, 2^. It has been believed that the predominant cause of POI in classic galactosemia is premature depletion of ovarian follicles (follicle depletion type of POI), resulting from insult to the ovary occurring early in life or even prenatally^3, 4^. Several mechanisms have been postulated to explain POI in these patients, including toxic effects of galactose and its metabolites on the ovary through the generation of reactive oxygen species (ROS)^5, 6^, aberrant function of follicle stimulating hormone (FSH) and FSH receptor due to glycosylation abnormalities^7^, deficiency of GALT leading to ovarian dysfunction^8^ and epigenetic mechanisms^9^, however an exact pathophysiology for this complication has not been elucidated.

Despite a galactose restricted diet, galactosemic patients have ambient plasma galactose ranging from 0.58–11.71 μmol/l (mean 2.72 μmol/l) versus 0.38–1.48 μmol/l in controls without galactosemia, plasma galactitol ranging from 9.28–15.9 μmol/l (mean 11.6 μmol/l) versus undetectable in normal controls, and red blood cell galactose 1-phosphate level ranging from 72–425 μmol/l (mean 161 μmol/l) equivalent to 1–4 mg/dl as compared to <1 mg/dl in non galactosemics^10^. This persistent elevation of galactose metabolites is explained by endogenous production of galactose which can range from 0.53–1.05 mg/kg/h^11^. Importantly, concentrations of galactose in pre-ovulatory follicular fluid have been shown to mirror plasma concentration in women without galactosemia^12^. In animal models, rats fed with a dietary excess of galactose had reduction in the number of spontaneous ovulations, diminished ovarian response to gonadotropin stimulation, and decreased litter size. In addition, female offspring of these rats demonstrated significant reduction in the number of oocytes^4, 13^. Therefore, research suggests that elevated plasma and thus follicular fluid levels of galactose and its metabolites is related to adverse reproductive outcomes such as alterations in embryo development and demonstration of epigenetic modifications passed on to subsequent generations^3, 4, 9, 13^.

Classic galactosemia, like other metabolic disorders such as diabetes^14^ and reproductive diseases including polycystic ovary syndrome, endometriosis, recurrent pregnancy loss and infertility^15^, has been associated with oxidative stress mediated by ROS^16^. In mouse^17–19^ and fly^20, 21^ models, exposure to high levels of dietary D-galactose was associated with negative long-term outcomes including neurodegeneration, cognitive disability, diminished immune response, and decreased lifespan that appear to be mediated by oxidative stress^22–26^. Furthermore, in galactosemic animal models lower than expected antioxidant activity was observed in tissues revealing that the insult caused by elevated levels of ROS are compounded by decreased protective machinery^21^. In fact, anecdotal reports demonstrated that galactosemic patients with poor dietary control displayed lower antioxidant activity and increased markers of oxidative stress^27, 28^. Interestingly, administration of antioxidants has shown potential to reverse the damages of galactose-dependent free radical generation in rat brain homogenates^29^. Oxidative stress mediated damaging effects have also been shown to alter reproductive function and ability in homozygous GALT gene-trapped mice pups^30^.

In the current study, we hypothesize that in some women with classic galactosemia POI results from follicle dysfunction due to toxic effects of D-galactose and its metabolites on oocyte quality mediated by oxidative stress. We choose to study the metaphase II spindle structure and chromosome alignment as markers of oocyte quality, as these are sensitive to alterations in the oocyte microenvironment^31–35^. However, as human oocytes are not readily available for research from patients with this rare disorder, we investigated the effects of D-galactose and its metabolites, galactose 1-phosphate and galactitol on mouse oocyte quality allowing for a close approximation to human response. We also examined the mechanisms through which D-galactose and its metabolites mediate follicle dysfunction in classic galactosemia including the ability of the oocytes exposed to these metabolites to fertilize, cleave and develop into blastocysts after *in-vitro* fertilization (IVF). Classic galactosemia patients in whom spontaneous pregnancies are reported, highlight the fact that the damage may not be absolute and that these women may stand to benefit from research that could protect or improve oocyte quality.

## Results

### D-galactose and its metabolites altered the oocyte spindle structure and chromosomal alignment

**Figure 2:**
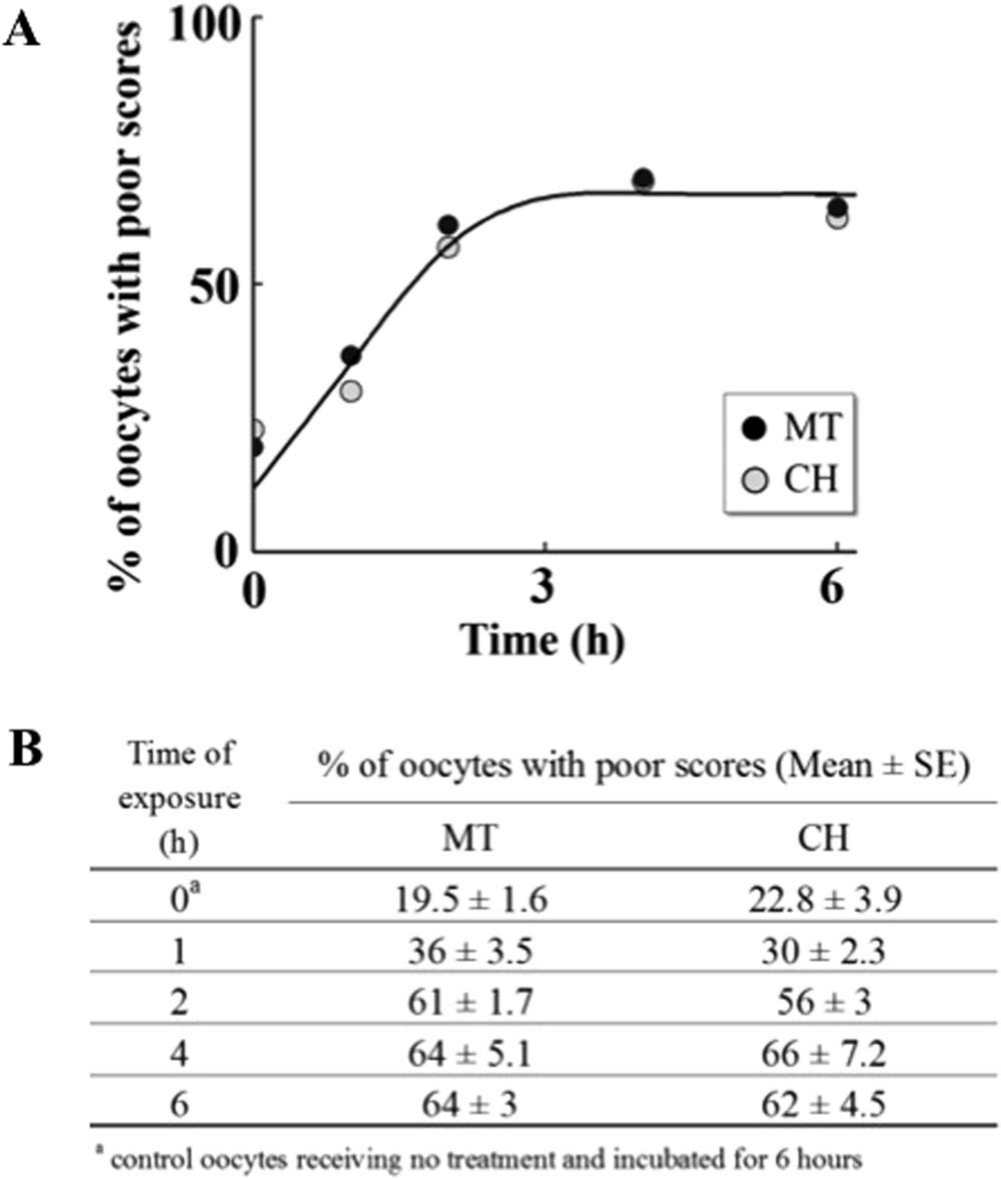
Time dependent effect of galactose 1-phosphate on oocyte quality. Percentage of oocytes with poor outcomes in MT structure and CH alignment at different incubation times (1, 2, 4 and 6 hours) when the oocytes without cumulus cells (n = 10 for each concentration) were incubated with fixed concentration of galactose 1-phosphate (0.1 mM) compared to control oocytes receiving no treatment and incubated for 6 hours (0 point on the Y axis) followed by indirect immunofluorescence staining. The table at the bottom presents the mean of percentage of oocytes with poor scores for each exposure time with the standard error of mean.

**Figure 3:**
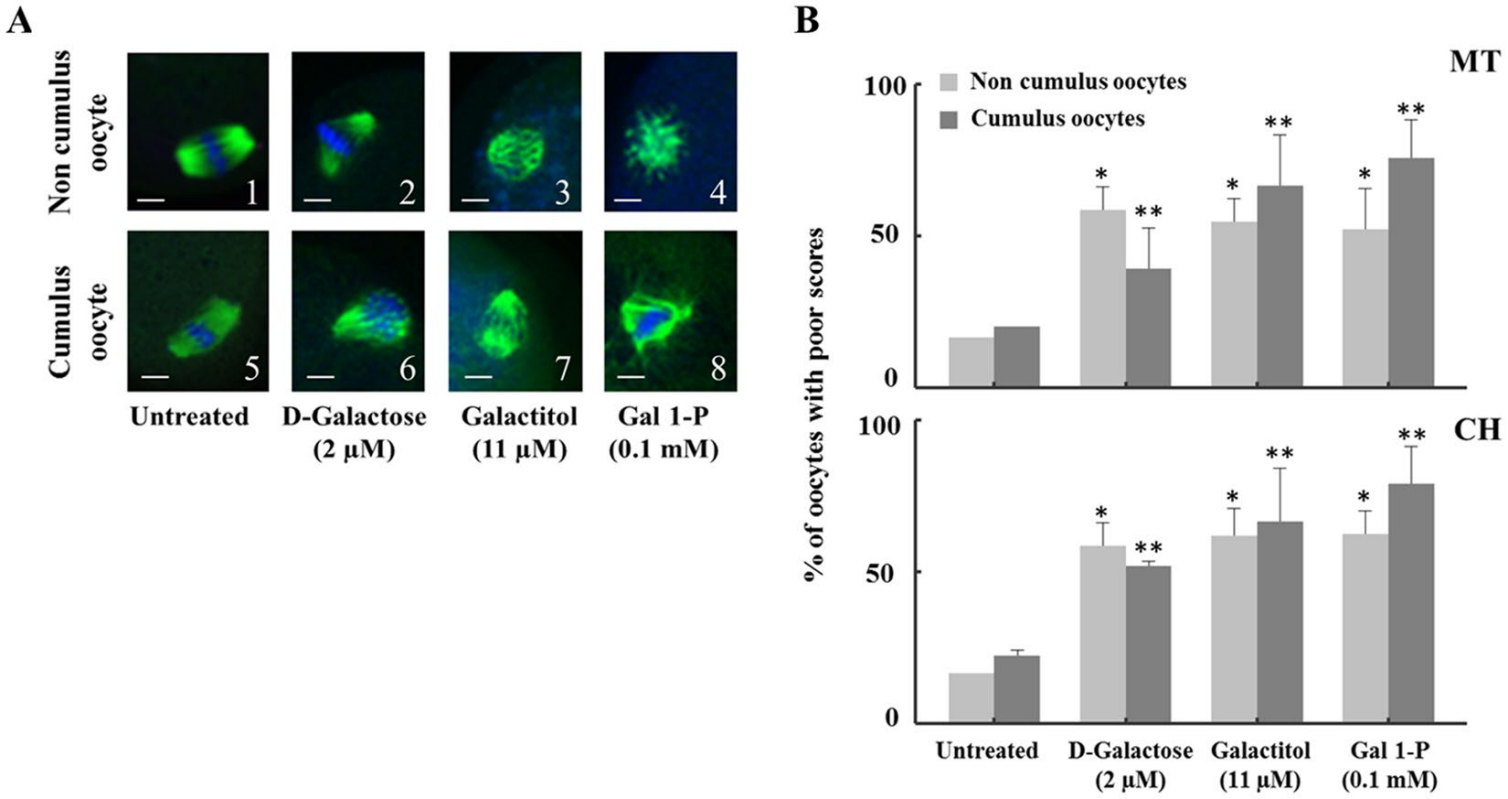
The effect of galactose and its metabolites on Metaphase II mouse oocyte spindles and chromosomes. (**A**) Representative confocal images of non-cumulus and cumulus metaphase II mouse spindles stained with β-tubulin antibody to visualize the microtubules (MT) (green) and counterstained with DAPI to visualize chromosomes (CH) (blue). After 4 hours of incubation, various abnormal configurations of spindles were observed when oocytes were exposed to D-galactose (**B**,**F**), galactitol (**C**,**G**) or Gal 1-P (galactose 1-phosphate) (**D**,**H**) compared to normal spindle shapes in untreated group (**A**,**E**) (n = 30/group). Scale bars: 1 pixel, 3 mm. Images shown are from a typical triplicated experiment. (**B**) The percentage of oocytes with poor scores in MT structure (upper panel) and CH alignment (lower panel) (120 cumulus and 120 without cumulus) in untreated oocytes compared to oocytes treated with galactose, galactitol and Gal 1-P (galactose 1-phosphate). Poor scores were significantly increased in oocytes exposed to galactose and its metabolites compared with controls in both MT and CH, indicated by *for oocytes without cumulus cells (p = 0.032 for MT and p = 0.05 for CH) and **for oocytes with cumulus cells (p = 0.04 for MT and p = 0.029 for CH). The experiment was conducted in triplicate.

To test whether D-galactose and its metabolites deteriorate mouse metaphase II oocyte quality, we investigated their effects on oocyte microtubule (MT) structure and chromosome alignment (CH) based on a well-established 1–4 scoring system^33, 34^ (see methods section for more details) and compared them to controls. To justify the desired time of incubation, we first investigated the time dependent effect by incubating oocytes with and without cumulus cells with D-galactose (2 µM), galactitol (11 µM) and galactose 1-phosphate (0.1 mM), levels corresponding to plasma concentrations of the metabolites present in classic galactosemia patients strictly compliant with diet, for 1, 2, 4, and 6 hours (n = 10 for each time interval); and the percentages of oocyte with poor scores were plotted as a function of time and compared to control oocytes receiving no treatment and incubated for 6 hours (n = 10) in the medium. The spindle morphology for the control oocytes remained almost the same over the 6-hour incubation period. In all treatments, the maximum degree of MT and CH alterations (60–85%) were reached at 4 hours and remained unaltered at longer time of incubation. Representative sample of the time dependent increase in the percentage of poor scores for MT and CH for exposure to galactose 1-phosphate is shown in Fig. 2. Based on these results, for all subsequent investigations an incubation time of 4 hours was chosen. We next investigated the effect of D-galactose and its metabolites in deteriorating oocyte quality by following the disruption of the MT structure and CH alignment. Figure 3A shows the representative confocal images of spindle morphology and chromosomal alignment from oocytes exposed to D-galactose and its metabolites compared to controls. Untreated oocytes without and with cumulus cells showed a symmetrical well-organized barrel shaped spindle structure (green) with chromosomes aligned along the spindle equatorial plate (blue) (Fig. 3A 1 & 5 respectively). Various abnormal configurations in MT and CH were noted after exposure to D-galactose, galactitol and galactose 1-phosphate (Fig. 3A 2–4 and 6–8). With exposure to D-galactose, the spindle poles are noted to pivot around the spindle-chromosomal attachments producing a “C” or “V” shape, whereas galactitol exposure produced a dilated “balloon” shaped spindle, and exposure to Gal-1-P produced a stellate configuration. Figure 3B shows the percentage of poor scores for MT (upper panel) and CH (lower panel) for oocytes without and with cumulus cells treated with galactose and its metabolites compared to controls. Oocytes without cumulus cells exposed to D-galactose and its metabolites had significantly increased poor scores (50–70%) compared with controls in both MT and CH (p = 0.032 for MT and p = 0.05 for CH). Comparison between controls and exposure groups in oocytes with cumulus cells also demonstrated increased poor scores with treatment with the three compounds for both MT and CH (p = 0.04, and 0.029 respectively) (Fig. 3B). Poor scores were similar in the oocytes with and without cumulus cells exposed to the three metabolites.

In parallel, exposure of fresh oocytes without (n = 120) and with (n = 120) cumulus cells to galactose and its metabolites led to similar damage to the spindle as seen with frozen oocytes. Experiments were repeated in triplicate (n = 10 per group) under identical conditions as above by incubating each group with D-galactose (2 µM), galactitol (11 µM) and galactose 1-phosphate (0.1 mM) and compared to untreated oocytes incubated for 4 hours. Representative confocal images of spindle morphology and chromosomal alignment from fresh oocytes without cumulus cells exposed to D-galactose and its metabolites compared to controls are presented in Supplementary Figure 1. The percentage of poor scores for MT and CH for oocytes exposed to galactose and its metabolites showed similar percentage of increased poor scores (55–70%) compared with controls in both MT and CH (p < 0.001 for both MT and CH). Collectively, exposure to galactose and its metabolites induced damage to the spindle to a similar degree in both fresh versus frozen oocytes, and this damage occurred independently of cumulus cells presence. Therefore, for all subsequent experiments only frozen oocytes were used.

**Figure 4:**
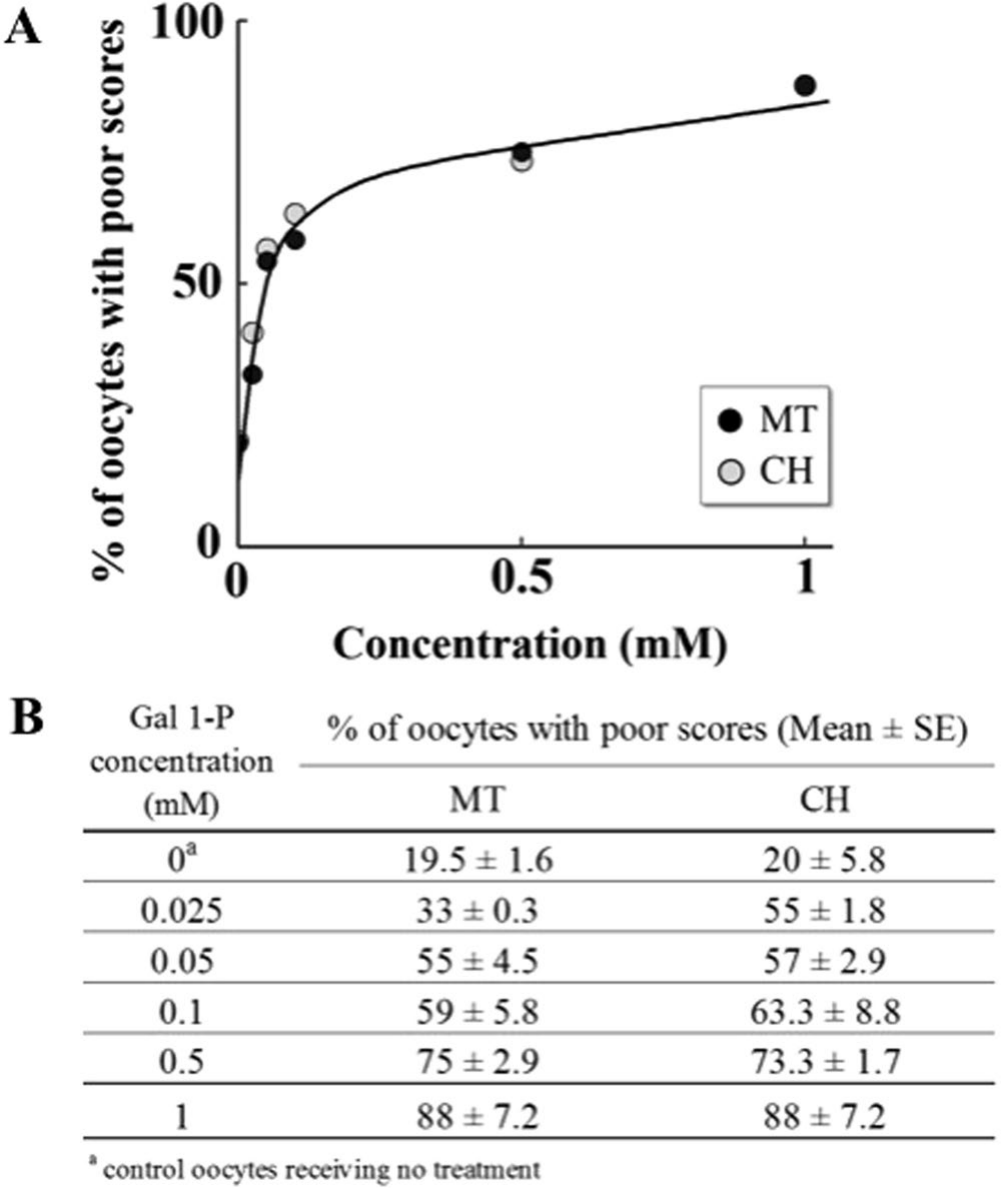
Concentration dependent effect of galactose 1-phosphate on oocyte quality. Percentage of oocytes with poor outcomes in MT structure and CH alignment, at 4 hours of incubation, when the oocytes without cumulus cells were incubated with increasing concentrations of galactose 1-phosphate (0.025 mM to 1 mM) {n = 10 for each concentration}, followed by indirect immunofluorescence staining. The table at the bottom presents the mean of percentage of oocytes with poor scores for each concentration with the standard error of mean.

To determine the effect of the concentration range that covers the levels of red blood cell galactose 1-phosphate in patients with classic galactosemia, we investigated the effects of increasing concentration of galactose 1-phosphate (0.025 mM to 1 mM), on oocyte quality after exposure for 4 hours (n = 10 for each concentration). As shown in Fig. 4A, poor oocyte quality was increased in a concentration dependent and saturable manner. Figure 4B summarizes the scores for MT structure and CH alignment.

To investigate whether the observed spindle damage was reversible, experiments were performed in two sets. In both sets, oocytes were exposed to 2 μM D-galactose, 11 μM galactitol or 0.1 mM galactose 1-phosphate (n = 10 for each metabolite) for four hours. Sibling control oocytes were incubated in medium without any treatment. In set 1, spindle and chromosomal morphology (MT and CH) were determined in exposed and control oocytes; while in set 2, control and exposed oocytes were thoroughly washed three times and incubated for 1 addition hour in HTF medium, followed by spindle evaluation. Oocytes washed after exposure to galactose and its metabolites were statistically compared to the controls (untreated oocytes and oocytes exposed to galactose and its metabolites). Untreated control oocytes washed three times and incubated additional hour in HTF media showed no significant alteration in oocyte quality. Collectively, the damage induced to the exposed oocyte spindles was irreversible, under our experimental condition, with similar damage observed in the two sets.

### D-galactose and its metabolites adversely affected oocyte cleavage and blastocyst development in embryos produced by *in-vitro* fertilization

**Figure 5:**
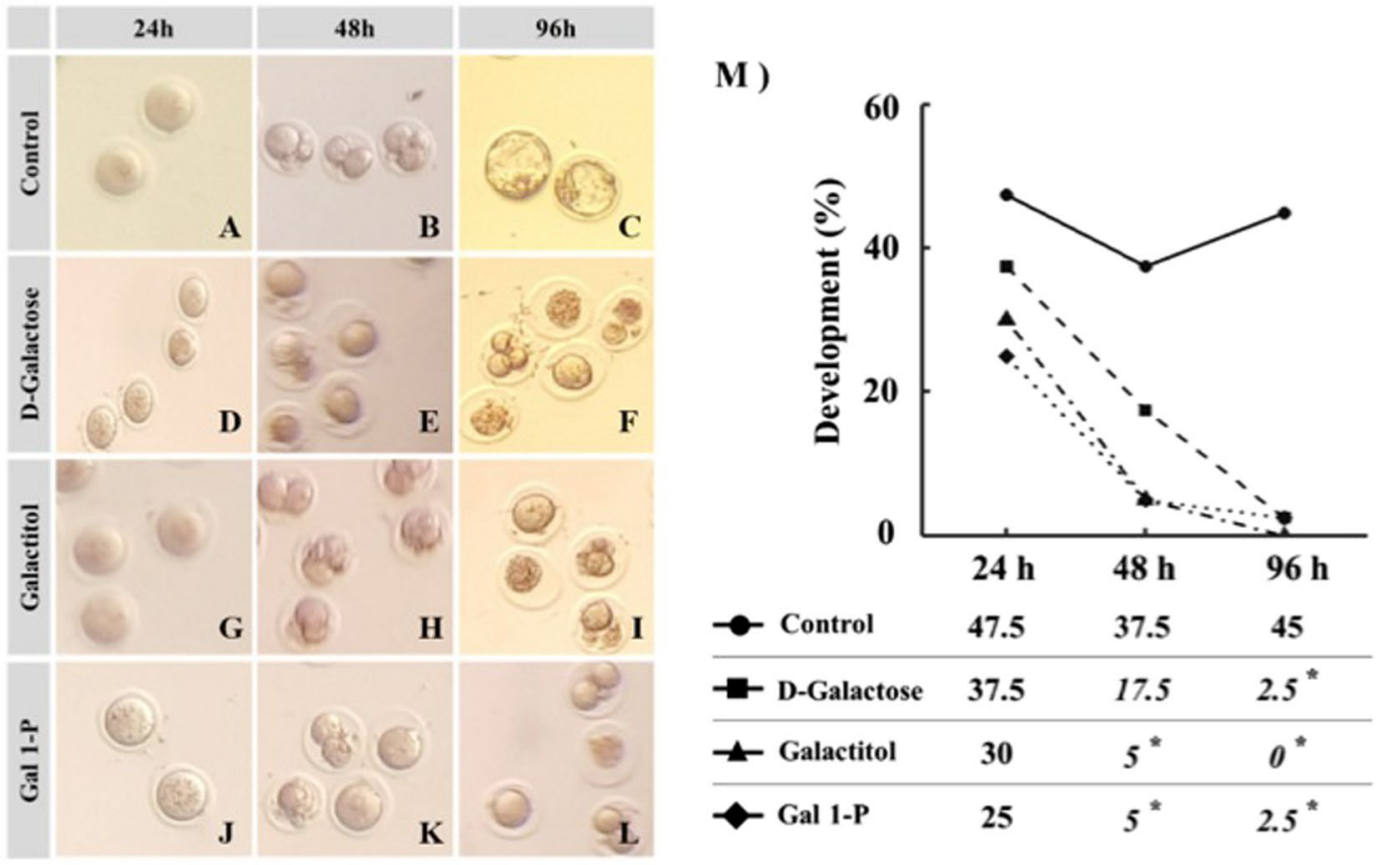
The effect of galactose and its metabolites on developmental competence of embryos generated by *in-vitro* fertilization. Images (**A**–**L**) represent images of embryo morphology at 24 hours (2-cell stage), 48 hours (8 cell stage) and 96 hours (blastocyst stage) after exposure to D-galactose (Images **D**–**F**), galactitol (Images **G**–**I**), and Gal 1-P (galactose 1-phosphate) (Images **J**–**L**), and untreated control (Images **A**–**C**). (**M**) The trends of changes in percentages of developed embryos at each stage compared to untreated controls.

The effects of D-galactose, galactitol and galactose 1-phosphate were then investigated on the cleavage and blastocyst rates after IVF (Fig. 5). At 24 hours post insemination, the majority of the oocytes exposed to galactose and its metabolites appeared granular and failed to fertilize, however the cleavage rate 24 hours post-fertilization was not statistically significant in the different exposure groups. The rates of embryo development at 48 hours were significantly lower in oocytes exposed to galactitol (5%) and galactose 1-phosphate (5%) as compared to control (37.5%). A similar trend was also observed in oocytes exposed to D-Galactose (17.5%) but did not reach statistical significance. Most treated zygotes exhibited fragmentation and a large perivitelline space. After 96 hours post-insemination, the rates of expanded blastocysts were significantly lower in all three treatment groups; D-galactose (2.5%), galactitol (0) and galactose 1-phosphate (2.5%) in comparison to controls (45%). The arrested embryos were highly fragmented and atretic.

### D-Galactose and its metabolites increased ROS generation in cumulus oocyte complex

**Figure 6:**
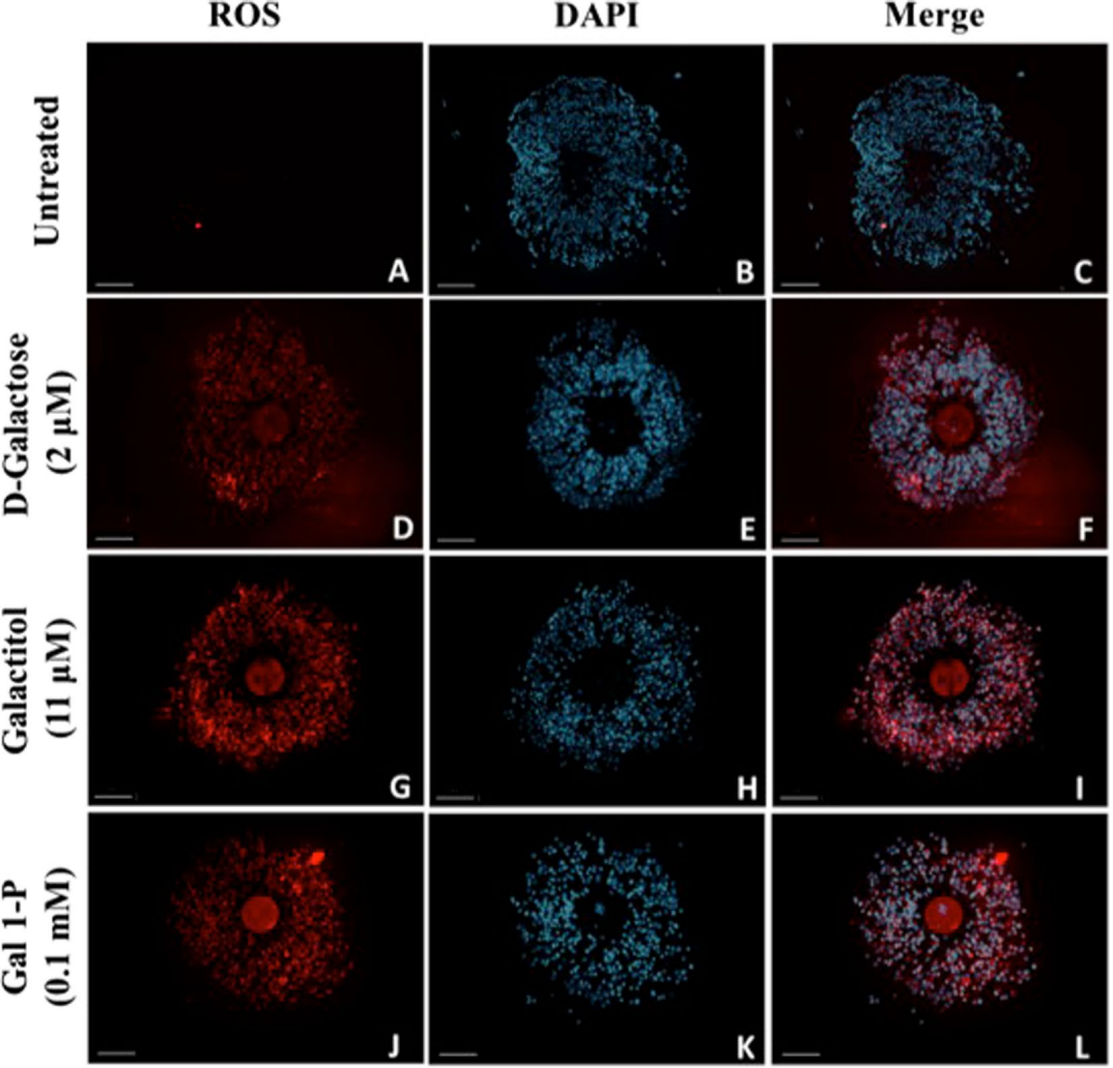
Evaluation of ROS generation. Images (**A**–**L**) represent images of intracellular ROS generation, 4′,6-diamidino-2-phenylindole (DAPI) fluorescence and merged images of ROS generation and DAPI of cumulus oocyte complex exposed to D-galactose (Images **D**–**F**), galactitol (Images **G**–**I**), and Gal 1-P (galactose 1-phosphate) (Images **J**–**L**), and untreated control (Images **A**–**C**). Scale bars: 100 μm. Images shown are from a typical experiment performed at least three times.

To further understand the mechanism of action behind galactose-induced damage to oocyte MT and CH, we evaluated ROS generation in the COC (Fig. 6). Treatment with D-galactose (Panel D–F), galactitol (Panel G–I) and galactose 1-phosphate (Panel J–L) led to increase in ROS generation in both the oocyte and the surrounding CCs compared to controls (Panel A–C) as indicated by increased ROS-mediated deep red fluorescence. Images of nuclear staining with DAPI (Panels B, E, H, K) and merged images of deep red fluorescence of ROS and DAPI staining (Panels C, F, I, L) were obtained to assess cell density. Treated oocytes demonstrated fluorescence in both the oocyte and CCs indicating that the ROS generation was higher in treated oocytes as compared to controls.

### D-Galactose and its metabolites increased apoptosis in the cumulus cells

**Figure 7:**
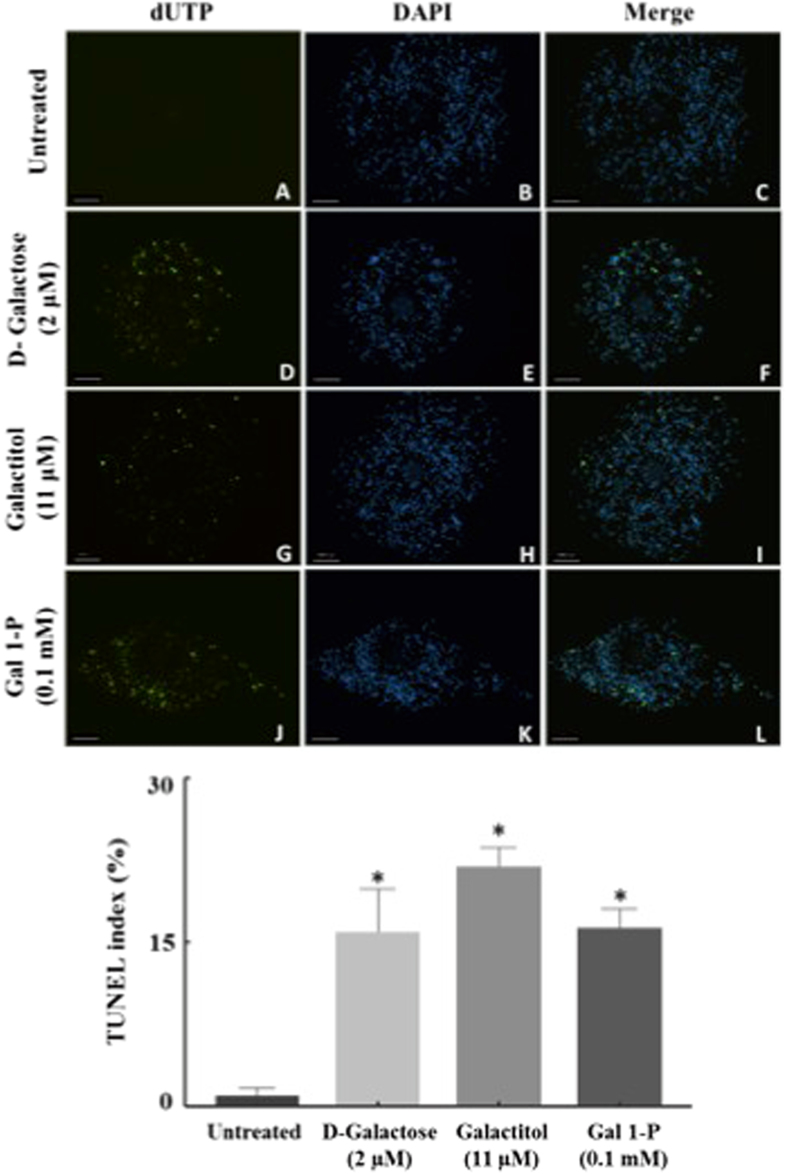
Detection of apoptosis by TUNEL in the cumulus-oocyte complex. Images (**A**–**L**) represent images of TUNEL assay (dUTP) (green), DAPI fluorescence (blue) and merged images of dUTP and DAPI in oocyte cumulus complex exposed to galactose (Images **D**–**F**), galactitol (Images **G**–**I**), and galactose 1-phosphate (Images **J**–**L**), compared to untreated control (Images **A**–**C**). Scale bars: 100 μm. Images shown are from a typical experiment performed at least three times. The number of TUNEL-positive cumulus cells were increased by D-galactose, galactitol and galactose 1-phosphate compared to no-treatment control. TUNEL Index was determined as the ratio of TUNEL-positive cells to total counted cell nuclei and presented as mean ± SEM (standard error of mean). *p < 0.05 compared to control.

We next observed the effect of D-galactose and its metabolites on oocyte and cumulus cell apoptosis by TUNEL staining, an indicator of the degree of DNA fragmentation (Fig. 7 upper panel). Nuclei were stained with DAPI (blue; Fig. 7B,E,H,K) and apoptotic cells were assessed (10x) with fluorescein-12-dUTP (green; Fig. 7A,D,G,J). Collectively, TUNEL index of COCs, calculated as percentage of apoptotic cells (TUNEL-positive nuclei) relative to the total number of the cells (DAPI-positive nuclei), showed a significant increase in mean percentage of TUNEL stained CCs (Fig. 7 lower panel) exposed to D-galactose (15.9% ± 3.9), galactitol (16.2% ± 1.7) and galactose 1-phosphate (21.8% ± 1.8) as compared to control (0.9% ± 0.7). Furthermore, our results indicated that D-galactose and its metabolites only mediated cumulus cell and not oocyte apoptosis.

### L-Galactose did not alter oocyte spindle structure, lead to generation of ROS, or cause apoptosis of cumulus cells

To exclude the possibility of an osmotic mechanism, experiments were performed with L-galactose. No significant increase in poor scores was observed in both MT and CH compared with controls in oocytes with and without cumulus exposed to L-galactose. Furthermore, exposure to L-galactose did not lead to significantly increased ROS generation. Similarly, L-Galactose had no effect on apoptosis of cumulus-oocyte complex cells as the TUNEL index did not differ significantly in L-Galactose treatment (6.1 ± 1.4) compared to control (0.9 ± 0.7; p = 0.88).

## Discussion

Here, we explore the effects of D-galactose and its metabolites on oocyte quality and function at plasma concentrations of patients compliant with a galactose restricted diet. The damage appears to be effected through ROS overproduction that mediates abnormalities in the spindle shape and therefore oocyte quality, and deterioration of the COC through the loss of the oocyte’s support. Our results suggested that D-galactose and its metabolites disturbed the parameters of oocyte quality, which were associated with significant decline in oocyte cleavage and blastocyst development after IVF. Treated oocytes showed increased ROS production that mediated the aforementioned damage. Furthermore, ROS mediated damage occurred independently of CCs, and in fact elevated ROS was related to loss of cumulus cell viability. This study provides the first direct evidence of the ROS mediated mechanism between classic galactosemia and oocyte quality and through an understanding of this mechanism provides the first platform to develop therapeutic interventions.

The mechanism of ROS mediated deterioration of oocyte quality could be through the generation of ROS through multiple pathways including abnormal galactose metabolism, mitochondrial damage, and decreased antioxidant machinery, or a combination of these processes. Our results show that damage to the oocyte was through a ROS mediated process but not through osmotic induced damage. Relatively very low concentration of D-galactose and its metabolites were used for our studies (Figs 3 and 5–7). In addition, exposure to L-galactose, a structural enantiomer of D-galactose that is not metabolized by living organisms, yet generates the same osmotic pressure in a solution, did not lead to any adverse effects on oocyte quality. Therefore, it is unlikely that deterioration in oocyte quality and CCs by D-galactose and its metabolites occurred through an osmotic activity based mechanism. In a small cohort of patients with galactosemia, elevated levels of free radicals caused increased activity of superoxide dismutase, an enzyme, which catalyzes the conversion of the toxic oxidant superoxide into hydrogen peroxide^36^. There is a growing body of evidence suggesting that accumulated D-galactose and its metabolites may lead to energy strain on cells by inhibiting key glycolytic enzymes, thus leading to mitochondrial stress, which independently creates ROS^37, 38^. Similarly, galactitol has also been shown to decrease antioxidants such as glutathione and ascorbic acid, strongly contributing to the oxidative stress in the cell^39^. This stress may make the oocyte more susceptible to ROS accumulation. The cellular antioxidant machinery provided from the surrounding CCs or intrinsically from the oocyte may be overcome by elevated ROS. Enhancement of ROS generation resulted in apoptosis of CCs, which was consistent with studies in animal models where galactose dose-dependently increased generation of ROS and caspase 3 in ovarian follicles^5^. Our results show that ROS mediated oocyte deterioration occurs independently of CCs, suggesting that the mechanism of CC apoptosis is highly likely to be the same process that ultimately compromises oocyte function.

Accumulation of ROS such as superoxide radical (O_2_^·^^−^), hydrogen peroxide (H_2_O_2_), singlet oxygen, and hydroxyl radicals (^·^OH) causes lipid and protein oxidation and subsequent cell damage^40^. Recent work in several laboratories has convincingly shown that ROS such as O_2_^·−^, H_2_O_2_, ·OH, HOCl, and peroxinitrite (ONOO^−^) can alter the oocyte quality in dose dependent fashion manifested by hypergranulated cytoplasm, absence of perivitelline space, and abnormal spindle dynamics^31, 35, 41–43^. Apparently, exposure to galactose and its metabolites, led to ROS mediated distortion of the spindle morphology resulting in deviation of the pole-to-pole axis and eventually breakage of the microtubule fibers and loss of chromosomal alignment. The process of the spindle damage is consistent with data from our lab and others that demonstrates that the process of spindle damage is likely through the disassembly of the spindle force balance and scaffolding proteins and that the process is irreversible^44^. Recent *in vitro* investigations have shown that H_2_O_2_ like ·OH, decreased the viability of cumulus cells at higher concentrations whereas other oxidants such as HOCl, and ONOO^−^, stripped the cumulus cells from the oocyte as well as dissolved the zona pellucida at low concentrations^31, 32^. Exposure to galactose metabolites demonstrated recognizable patterns of spindle morphologic change, which was noted in other inflammatory conditions such as diabetes, and exposure to oxidants such as ONOO^−^ and toxins such as nicotine and bisphenol-A^44–46^. Therefore, oocyte damage in classic galactosemia, similarly to other inflammatory disease, may be mitigated by therapies aimed at elimination of galactose metabolites, thus decreasing ROS or increasing antioxidant activity.

In our study, elevated galactose 1-phosphate showed concentration dependent damage to the oocyte spindle, and even levels that are considered acceptable in patients on dietary restriction were damaging. Consistent with our study, it has been proposed that elevated galactose 1-phosphate may be a major pathogenic factor in this disorder^47, 48^. However, elimination of galactose 1-phosphate cannot be achieved by dietary restriction alone. Endogenous production of galactose and subsequent conversion to galactose 1-phosphate is not amenable to dietary therapy^11, 49^. In addition, galactose 1-phosphate can be derived from non-dairy sources^50^. Therefore, in addition to dietary therapy, elimination of galactose 1-phosphate levels by inhibition of GALK may be a potential therapeutic target for classic galactosemia^51, 52^. Quantitative high-throughput screening is being utilized to search for compounds with selective GALK inhibitor activity^51–53^. Another therapeutic approach is reduction in galactitol levels by aldose reductase inhibitors, which have been used successfully in diabetic patients^39, 54^. Similarly, the role of enzymatic and non-enzymatic antioxidant defense strategies by medication and/or nutrition in mitigating multiple other long term complications from galactosemia including cataracts, retinopathy, decreased bone mass and brain abnormalities need to be explored^55, 56^. In the D. melanogaster model of classic galactosemia, free radical scavengers, which mimic the action of superoxide dismutase, have been shown to modify both acute and long-term outcome survival and development^56^. These manganese-containing porphyrin compounds showed little toxicity, and thus have considerable potential as therapeutic compounds^56^. Similarly, purple sweet potato color, a plant extract rich in acetylated anthocyanins, which have been shown to quench free radical production, has also been suggested as a potential therapeutic option^57^.

Our results indicate that exposure to galactose and its metabolites induces a cellular state where ROS production exceeds the oocyte and surrounding cumulus cells ability to neutralize their harmful effects. Excessive accumulation of ROS overwhelms cellular defense machinery and not only induces cellular toxicity through lipid peroxidation, DNA and RNA damage, but also regulate altered gene expression through both genetic and epigenetic cascades in patients with classic galactosemia^58, 59^. Indeed, previous studies have noted that exposure to high levels of galactose may lead to a transgenerational effects on follicle development^4, 13^. Epigenetic modification of two genes, aplysia ras homolog I (ARH1) and growth differentiation factor-9 (GDF9) have been considered as plausible targets of galactose toxicity^3, 9^. We observed that exposure to galactose and its metabolites led to disruption of the functional capacity of oocytes to develop into embryos. Whether this effect is mediated through ROS, affectation of the metabolic processes in the oocytes, through genetic and epigenetic modification or a combination of all, needs to be explored.

In conclusion, our results demonstrate that galactose exposure mediated by ROS is the major cause of adverse reproductive outcomes in patients with classic galactosemia. These studies are important as they provide a deviation and likely a correction to the previous ideology on the pathophysiology of classic galactosemia in which follicular depletion is an unwavering end. The concept of ROS mediated follicular dysfunction, opens the door to potential therapeutic options in the form of further reducing the plasma level of galactose and its metabolites and/or administering antioxidants, which may provide potential ways to extend the window of fertility for women with classic galactosemia.

## Materials and Methods

The experimental procedures used for live animal care and handling were approved by Institutional Animal Care and Use Committee at Wayne State University (Protocol #A 11-01-15) and Embryotech Laboratories Inc., Haverhill, MA, USA. The methods were carried out in accordance with the approved guidelines.

### Materials

All the materials used were of the highest grade of purity. No further purification was necessary. D-galactose (G5388), L-galactose (G7134), galactose 1-phosphate (G0380), galactitol (D0256), human tubular fluid (HTF) medium, M2 medium, anti-α tubulin antibody, fluorescein isothiocyanate (FITC) conjugate anti-goat antibody, 4′,6′-diamino-2-phenylindole (DAPI), 1% Bovine Serum Albumin (BSA), 0.1% M Glycine, and 0.1% Triton X- 100 were obtained from Sigma–Aldrich (St. Louis, MO, USA). Normal Goat Serum (2%) was from Invitrogen (Grand Island, NY), 0.2% Powder Milk (Nestle) from grocery and anti-fade agent was obtained from Biomedia, CA. Cellular reactive oxygen species (ROS) detection assay kit (ab186029, Abcam, Cambridge, United Kingdom) and *In Situ* Cell Death Detection kit, AP (11684795910, Roche Applied Science, Penzberg, Germany) were procured from respective commercial sources.

### Methods

Superovulation and oocyte retrieval was performed as previously described with modifications^60–62^. Briefly, three-week-old B_6_C_3_F_1_ mice (6101F, Envigo, Indianapolis, IN, USA) were superovulated. Three week old mice are generally selected for superovulation as the number and quality of oocytes from these animals is higher as compared to females past puberty^63^. Pregnant mare’s serum gonadotropin (PMSG) and hCG (Sigma, Saint Louis, MO) (7.5 IU each) was administered intraperitoneally 48–52 hours apart. Metaphase II oocytes were retrieved from oviductal ampullae 18 hours after hCG injection. The cumuli were incubated with 0.1% hyaluronidase (w/v) in M2 medium for 2–3 min at 37 °C to release oocytes. The denudation process was facilitated by removing all cumulus-corona cells with a narrow bore pulled glass Pasteur pipet. Oocytes were rinsed in M2 medium, graded to confirm normal morphology and divided into two groups. For experiments with fresh oocytes (n = 120), oocytes were transferred to HTF medium pre-equilibrated with 5% CO_2_ in air at 37 °C and were subsequently randomly assigned to test (treatment with galactose, galactose 1-phosphate, galactitol) and control groups. Rest of the metaphase II oocytes (with and without cumulus cells) (n = 300) were cryopreserved in straws using ethylene glycol-based slow freeze cryopreservation protocol (Embryotech Laboratories, Inc. Haverhill, MA, USA). We choose to use frozen-thawed oocytes as large numbers of oocytes were used for the different experiments requiring multiple time consuming steps and use of frozen oocytes was more convenient. We have demonstrated that fresh and frozen oocytes yield similar and reproducible results, specifically when incubated in media for at least 60 minutes allowing the spindles to repolymerize to normal architecture and cumulus cells maintain viability^31, 35, 42, 60, 61, 64^. Our conclusion is also supported by previous studies and confirmed by current experiments that demonstrate that incubating thawed oocytes in media for at least 60 minutes repolymerizes the spindle and frozen cumulus cells maintain their viability and DNA integrity after thawing^65, 66^ and showed similar damage to the spindle after exposure to galactose and its metabolites.

### Exposure of metaphase II mouse oocytes to D-galactose and its metabolites

The study consisted of the following two experimental sets: 1) the effect of D-galactose and its metabolites on metaphase II mouse oocytes without cumulus cells (n = 120); 2) the effect of D-galactose and its metabolites on metaphase II mouse oocytes with cumulus cells (n = 120). Oocytes with cumulus cells were used to investigate whether cumulus cells provided any protection from the effects of D-galactose and its metabolites on oocyte quality. Oocytes with and without cumulus cells were thawed and transferred from straws to phosphate buffer saline (Dulbecco PBS) and washed for 3 minutes to remove cryoprotectant. Oocytes were then transferred to HTF media and incubated at 37 °C and 5% carbon dioxide (CO_2_) for 60 minutes to allow spindle repolymerization and attainment of normal oocyte architecture. The oocytes were then screened for the presence of the polar body confirming their Metaphase II stage. Immature oocytes or those that displayed disrupted zona pellucida were discarded.

In triplicate experiments, oocytes with and without cumulus cells (n = 10 oocytes in each group) were exposed to 2 μM D-galactose, 11 μM galactitol or 0.1 mM galactose 1-phosphate for four hours where maximum effects on the oocyte spindle were observed. The concentrations of the metabolites were selected based on plasma levels of patients with classic galactosemia, strictly compliant with a galactose restricted diet^10^. Untreated oocytes with and without cumulus cells served as controls.

To confirm that the damage to the spindle after exposure to galactose and its metabolites was similar between fresh versus frozen oocytes, some of the above experiments were repeated utilizing fresh oocytes under identical conditions. More details (number of oocytes and the experiments performed) are presented in the result section.

### Immunofluorescence staining and fluorescence microscopy

All the exposed and control oocytes were fixed in a solution prepared from 2% formaldehyde and 0.2% Triton X-100 for 30 minutes. The fixed oocytes were treated with blocking solution (PBS, 0.2% Powdered Milk, 2% Normal Goat Serum, 1% Bovine Serum Albumin (BSA), 0.1 M Glycine and 0.1% Triton X-100) for 30 minutes, and then washed with PBS for 3 minutes. Indirect immunofluorescence staining was then performed by incubating oocytes in mouse primary (anti-α tubulin) antibody for 60 minutes and secondary (FITC) conjugated anti-goat antibody for 30 minutes. The chromosomes were stained using DAPI and incubated for 15 minutes. Stained oocytes were loaded into anti-fade agent (Biomedia, CA, USA) on slides with two etched rings and cover slips placed using transparent nail varnish. Slides were stored at −20 °C and protected from light until they were evaluated for more details by confocal microscopy.

### Confocal microscopy, assessment of microtubules and chromosomal alignment

Slides were examined with the Axiovert 25 inverted microscope (Zeiss, Thornwood, NY) using DAPI (blue) and FITC (green) fluorescent filters with excitation and emission wavelengths of 358 and 461 nm, and 596 and 613 nm, respectively. Confocal images were obtained utilizing a Zeiss LSM 510 META NLO (Zeiss, Germany) microscope. Oocytes were localized using a 10x magnification lens and spindle alterations assessed using 40x oil immersion lens. The MT was stained fluorescent green, which was distinct from the fluorescent blue staining of the chromosomes. Following completion of the experiments each oocyte was closely examined for spindle status. Three blinded observers scored alterations in the microtubule structure (MTs) and chromosomal alignment (CH) compared with controls based on a previously published scoring system using comprehensive evaluation of spindle images obtained by immunofluorescence and confocal 3-dimensional reconstruction^33, 34^. Scores of 1–4 were assigned for both MT and CH alterations, with scores 1 and 2 representing good scores meaning microtubules were organized in a barrel-shape, formed by organized microtubules traversing from one pole to another and chromosomes were arranged in a compact metaphase plate at the equator of the spindle. Scores of 3 and 4 signified poor scores and consisted of spindle length reduction, disorganization and/or complete spindle absence, and chromosome dispersion or aberrant condensation appearance.

### *In-vitro* fertilization and evaluation of embryo development

Next, oocytes exposed to galactose and its metabolites as well as untreated controls were subjected to IVF (Embryotech Laboratories, Inc. Haverhill, MA, USA) and embryo development was examined. In total, 160 oocytes were used {n = 40, for each of the 4-groups; untreated oocytes, oocytes exposed to galactose (2 μM), galactitol (11 μM), and galactose 1-phosphate (0.1 mM)}. Metaphase II mouse oocytes were harvested from 3 weeks (21–24 day) old super ovulated hybrid B_6_C_3_F_1_ female mice (6101 F, Envigo, Indianapolis, IN, USA)^62^. The cumulus oocyte complexes were placed in a 0.5 ml drop of 0.1% hyaluronidase (w/v) to remove cumulus cells, washed three times and gradually equilibrated with HTF medium and exposed to galactose (2 μM), galactitol (11 μM) and galactose 1-phosphate (0.1 mM) and untreated controls. After incubation for four hours, the oocytes were washed twice with HTF (500 μL) and placed in global medium (LGGG-100, LifeGlobal, Guilford, CT, USA). Oocytes were then incubated for 2-hours with capacitated mouse sperm (one million sperms/ml) obtained by epididymal extraction from B_6_D_2_F_1_ male mice (6301 M, Envigo, Indianapolis, IN, USA) (40 oocytes per fertilization drop) as previously reported^62^. The inseminated oocytes were removed and rinsed in global medium and placed in 12 µl droplets (10 gametes per drop) covered with prewarmed mineral oil in a labeled well of the 60-well mini-tray Terasaki plate (163118, Nunc, Thermo Scientific, Waltham, MA, USA) and then cultured at 37 °C, in a humidified atmosphere of 5% CO_2_, for 3 days. The embryos were evaluated under an Olympus SZ61 dissecting microscope at up to 45x magnification at 24, 48 and 96 hours. Fertilization was assessed at 48-hours after IVF. The embryos were counted at the end of the culture period; 96–100 hours post fertilization, in order to determine blastocyst development^62^. The cleavage stage and blastocyst embryo quality was graded by embryologists, based on subjective assessment as previously described^67, 68^. The cleavage stage embryos were graded based on blastomere number, symmetry, and the presence of nuclear fragments. For the blastocysts, degree of expansion of blastocoel, the quality inner cell mass and the quality of trophectoderm was taken into account.

### Detection of ROS generation *in situ*

Generation of ROS was evaluated using the Cellular Reactive Oxygen Species Detection Assay Kit (ab186029, Abcam, Cambridge, United Kingdom). Briefly, the COCs exposed to D-galactose, galactitol and galactose 1-phosphate for 4 hours and controls (n = 30 in each group) were incubated with 100 µl of ROS Deep Red working solution in 5% CO_2_, 37 °C incubator for 60 minutes. The cells were fixed and with 2% formaldehyde and permeabilization with 0.2% Triton X-100 for 30 minutes and the nuclei of these cells were stained with DAPI. Appropriate positive controls were included in the test. The oocytes were subsequently mounted in anti-fade agent and images of ROS-mediated deep red fluorescence were taken by an independent examiner using a Nikon Eclipse 90i epifluorescence microscope and analyzed by NIS-Element (Nikon, Shinagawa-Ku, Tokyo, Japan).

### Detection of apoptosis in cumulus-oocyte complex

DNA fragmentation was assessed by the *in situ* Terminal Deoxynucleotidyl Transferase-mediated dUTP-biotin nick end labeling (TUNEL) technique per the *In Situ* Cell Death Detection kit, AP (11684795910, Roche Applied Science, Penzberg, Germany) manual. Briefly, the COCs exposed to D-Galactose, Galactitol and Galactose 1-phosphate for 4 hours and control oocytes were fixed with 2% formaldehyde and permeabilized using 0.2% Triton X-100 for 30 minutes. Next, COCs were labeled with the TUNEL reaction mixture for 60 min at 37 °C. The nuclei of these cells were stained with DAPI and the COCs were mounted on slides in anti-fade agent. Fluorescein-labeled DNA, an indication of DNA fragmentation, was evaluated for n = 10 COCs per treatment group by fluorescence microscopy by an independent examiner blinded to the exposure groups. The TUNEL index of COCs was calculated as percentage of apoptotic cells (TUNEL-positive nuclei) relative to the total number of the cells (DAPI-positive nuclei).

### Experiments with L-galactose as osmotic control

Oocytes with and without cumulus cells (n = 10), in triplicates, were exposed to L-galactose (2 μM) and microtubule and chromosomal alignment was assessed as described above. Similarly, ROS generation was evaluated using the Cellular Reactive Oxygen Species Detection Assay Kit (ab186029, Abcam, Cambridge, United Kingdom) and apoptosis of both cumulus cells and oocytes were performed using the TUNEL assay per the *In Situ* Cell Death Detection kit, AP (11684795910, Roche Applied Science, Penzberg, Germany) using the same methodology as for the other metabolites.

### Statistical Analysis

Statistical analyses were performed using Statistical Package for the Social Sciences (SPSS) version 22.0. Comparisons of percentage of oocytes with poor scores (scores 3 and 4) for MT and CH between control and treatment groups as well as the effect of different treatment on TUNEL indices were made using One-Way ANOVA with Tukey post hoc testing, and comparisons between oocytes without and with cumulus cells for each treatment were performed with Student t-test. The statistical significance of treatments on development capacity of fertilized oocytes was assessed by two-tailed Fisher exact test. P < 0.05 was considered significant for all statistical tests.

## Electronic supplementary material

Supplementary Figure 1

## References

[1] Rubio-Gozalbo ME (2010). Gonadal function in male and female patients with classic galactosemia. Human reproduction update.

[2] Forges T, Monnier-Barbarino P, Leheup B, Jouvet P (2006). Pathophysiology of impaired ovarian function in galactosaemia. Human reproduction update.

[3] Liu G (2006). Dietary galactose inhibits GDF-9 mediated follicular development in the rat ovary. Reproductive toxicology (Elmsford, N.Y.).

[4] Chen YT, Mattison DR, Feigenbaum L, Fukui H, Schulman JD (1981). Reduction in oocyte number following prenatal exposure to a diet high in galactose. Science (New York, N.Y.).

[5] Banerjee S (2012). Ovotoxic effects of galactose involve attenuation of follicle-stimulating hormone bioactivity and up-regulation of granulosa cell p53 expression. PloS one.

[6] Bandyopadhyay S (2003). Galactose toxicity in the rat as a model for premature ovarian failure: an experimental approach readdressed. Human reproduction (Oxford, England).

[7] Gubbels CS (2011). FSH isoform pattern in classic galactosemia. Journal of inherited metabolic disease.

[8] Berry GT (2008). Galactosemia and amenorrhea in the adolescent. Ann N Y Acad Sci.

[9] Lai K (2008). ARHI: A new target of galactose toxicity in Classic Galactosemia. Bioscience hypotheses.

[10] Ning C, Segal S (2000). Plasma galactose and galactitol concentration in patients with galactose-1-phosphate uridyltransferase deficiency galactosemia: determination by gas chromatography/mass spectrometry. Metabolism: clinical and experimental.

[11] Berry GT (1995). Endogenous synthesis of galactose in normal men and patients with hereditary galactosaemia. Lancet (London, England).

[12] Jozwik M, Jozwik M, Teng C, Battaglia FC (2007). Concentrations of monosaccharides and their amino and alcohol derivatives in human preovulatory follicular fluid. Molecular human reproduction.

[13] Swartz WJ, Mattison DR (1988). Galactose inhibition of ovulation in mice. Fertility and sterility.

[14] Wang Q (2009). Maternal diabetes causes mitochondrial dysfunction and meiotic defects in murine oocytes. Molecular endocrinology (Baltimore, Md.).

[15] Agarwal A, Aponte-Mellado A, Premkumar BJ, Shaman A, Gupta S (2012). The effects of oxidative stress on female reproduction: a review. Reproductive biology and endocrinology: RB&E.

[16] Al-Essa M, Dhaunsi GS, Al-Qabandi W, Khan I (2013). Impaired NADPH oxidase activity in peripheral blood lymphocytes of galactosemia patients. Experimental biology and medicine (Maywood, N.J.).

[17] Wei H (2005). Behavioural study of the D-galactose induced aging model in C57BL/6J mice. Behavioural brain research.

[18] Cui X (2006). Chronic systemic D-galactose exposure induces memory loss, neurodegeneration, and oxidative damage in mice: protective effects of R-alpha-lipoic acid. Journal of neuroscience research.

[19] Long J (2007). D-galactose toxicity in mice is associated with mitochondrial dysfunction: protecting effects of mitochondrial nutrient R-alpha-lipoic acid. Biogerontology.

[20] Jordens RG, Berry MD, Gillott C, Boulton AA (1999). Prolongation of life in an experimental model of aging in Drosophila melanogaster. Neurochemical research.

[21] Cui X (2004). D-galactose-caused life shortening in Drosophila melanogaster and Musca domestica is associated with oxidative stress. Biogerontology.

[22] Shang YZ, Gong MY, Zhou XX, Li ST, Wang BY (2001). Improving effects of SSF on memory deficits and pathological changes of neural and immunological systems in senescent mice. Acta pharmacologica Sinica.

[23] Shen YX (2002). Melatonin reduces memory changes and neural oxidative damage in mice treated with D-galactose. Journal of pineal research.

[24] Song X, Bao M, Li D, Li YM (1999). Advanced glycation in D-galactose induced mouse aging model. Mechanisms of ageing and development.

[25] Yelinova V (1996). Studies of human and rat blood under oxidative stress: changes in plasma thiol level, antioxidant enzyme activity, protein carbonyl content, and fluidity of erythrocyte membrane. Biochemical and biophysical research communications.

[26] Kowluru RA, Kern TS, Engerman RL, Armstrong D (1996). Abnormalities of retinal metabolism in diabetes or experimental galactosemia. III. Effects of antioxidants. Diabetes.

[27] Schulpis KH, Michelakakis H, Tsakiris T, Tsakiris S (2005). The effect of diet on total antioxidant status, erythrocyte membrane Na+, K+-ATPase and Mg2+-ATPase activities in patients with classical galactosaemia. Clinical nutrition (Edinburgh, Scotland).

[28] Schulpis KH, Papassotiriou I, Tsakiris S (2006). 8-hydroxy-2-desoxyguanosine serum concentrations as a marker of DNA damage in patients with classical galactosaemia. Acta paediatrica (Oslo, Norway: 1992).

[29] Tsakiris S, Carageorgiou H, Schulpis KH (2005). The protective effect of L-cysteine and glutathione on the adult and aged rat brain (Na+, K+)-ATPase and Mg2+-ATPase activities in galactosemia *in vitro*. Metabolic brain disease.

[30] Tang M (2014). Subfertility and growth restriction in a new galactose-1 phosphate uridylyltransferase (GALT) - deficient mouse model. European journal of human genetics: EJHG.

[31] Banerjee J (2013). Peroxynitrite affects the cumulus cell defense of metaphase II mouse oocytes leading to disruption of the spindle structure *in vitro*. Fertility and sterility.

[32] Banerjee J, Maitra D, Diamond MP, Abu-Soud HM (2012). Melatonin prevents hypochlorous acid-induced alterations in microtubule and chromosomal structure in metaphase-II mouse oocytes. Journal of pineal research.

[33] Choi WJ (2007). Oxidative stress and tumor necrosis factor-alpha-induced alterations in metaphase II mouse oocyte spindle structure. Fertility and sterility.

[34] Banerjee J (2012). IL-6 and mouse oocyte spindle. PloS one.

[35] Goud AP, Goud PT, Diamond MP, Gonik B, Abu-Soud HM (2008). Reactive oxygen species and oocyte aging: role of superoxide, hydrogen peroxide, and hypochlorous acid. Free radical biology & medicine.

[36] Vilaseca-Busca MA (2002). [Abnormal antioxidant system in inborn errors of intermediary metabolism]. Revista de neurologia.

[37] Aguer C (2011). Galactose enhances oxidative metabolism and reveals mitochondrial dysfunction in human primary muscle cells. PloS one.

[38] Liu G, Hale GE, Hughes CL (2000). Galactose metabolism and ovarian toxicity. Reproductive toxicology (Elmsford, N.Y.).

[39] Meyer WR (1992). Aldose reductase inhibition prevents galactose-induced ovarian dysfunction in the Sprague-Dawley rat. Am J Obstet Gynecol.

[40] Halliwell B, G. J. *Free radicals in biology and medicine.* 1–20 (Clarendon Press, Oxford, 1989).

[41] Goud PT (2014). Dynamics of nitric oxide, altered follicular microenvironment, and oocyte quality in women with endometriosis. Fertility and sterility.

[42] Shaeib F, Banerjee J, Maitra D, Diamond MP, Abu-Soud HM (2013). Impact of hydrogen peroxide-driven Fenton reaction on mouse oocyte quality. Free radical biology & medicine.

[43] Khan SN (2015). Diffused Intra-Oocyte Hydrogen Peroxide Activates Myeloperoxidase and Deteriorates Oocyte Quality. PloS one.

[44] Khan SN (2016). Peroxynitrite deteriorates oocyte quality through disassembly of microtubule organizing centers. Free radical biology & medicine.

[45] Wang Q, Moley KH (2010). Maternal diabetes and oocyte quality. Mitochondrion.

[46] Zenzes MT, Bielecki R (2004). Nicotine-induced Disturbances of Meiotic Maturation in Cultured Mouse Oocytes: Alterations of Spindle Integrity and Chromosome Alignment. Tobacco induced diseases.

[47] Robertson, A., Singh, R. H., Guerrero, N. V., Hundley, M. & Elsas, L. J. Outcomes analysis of verbal dyspraxia in classic galactosemia. *Genetics in medicine: official journal of the American College of Medical Genetics* **2**, 142–148, doi:10.109700125817-200003000-00005 (2000). 10.1097/00125817-200003000-00005 11397328

[48] Berry, G. T. In *GeneReviews*(*R*) (eds Pagon, R. A. *et al*.) (University of Washington, Seattle. All rights reserved., 1993).

[49] Berry GT (2004). The rate of de novo galactose synthesis in patients with galactose-1-phosphate uridyltransferase deficiency. Molecular genetics and metabolism.

[50] Acosta PB, Gross KC (1995). Hidden sources of galactose in the environment. European journal of pediatrics.

[51] Lai K, Boxer MB, Marabotti A (2014). GALK inhibitors for classic galactosemia. Future medicinal chemistry.

[52] Tang M, Odejinmi SI, Vankayalapati H, Wierenga KJ, Lai K (2012). Innovative therapy for Classic Galactosemia - tale of two HTS. Molecular genetics and metabolism.

[53] Wierenga KJ, Lai K, Buchwald P, Tang M (2008). High-throughput screening for human galactokinase inhibitors. Journal of biomolecular screening.

[54] Oka M, Kato N (2001). Aldose reductase inhibitors. Journal of enzyme inhibition.

[55] Ramana BV, Raju TN, Kumar VV, Reddy PU (2007). Defensive role of quercetin against imbalances of calcium, sodium, and potassium in galactosemic cataract. Biological trace element research.

[56] Jumbo-Lucioni PP (2014). Manganese-based superoxide dismutase mimics modify both acute and long-term outcome severity in a Drosophila melanogaster model of classic galactosemia. Antioxidants & redox signaling.

[57] Timson DJ (2014). Purple sweet potato colour–a potential therapy for galactosemia?. International journal of food sciences and nutrition.

[58] Coss KP (2014). Systemic gene dysregulation in classical Galactosaemia: Is there a central mechanism?. Molecular genetics and metabolism.

[59] Coman DJ (2010). Galactosemia, a single gene disorder with epigenetic consequences. Pediatric research.

[60] Goud PT, Goud AP, Diamond MP, Gonik B, Abu-Soud HM (2008). Nitric oxide extends the oocyte temporal window for optimal fertilization. Free radical biology & medicine.

[61] Goud AP, Goud PT, Diamond MP, Abu-Soud HM (2005). Nitric oxide delays oocyte aging. Biochemistry.

[62] Behringer, R., Gertsenstein, M., Nagy, K. N., Nagy, A. *Manipulating the Mouse Embryo: A Laboratory Manual* Fourth edn (Cold Spring Harbor Laboratory Press, 2014).

[63] Hoogenkamp H, Lewing P (1982). Superovulation in mice in relation to their age. The Veterinary quarterly.

[64] Goud AP, Goud PT, Diamond MP, Gonik B, Abu-Soud HM (2006). Activation of the cGMP signaling pathway is essential in delaying oocyte aging in diabetes mellitus. Biochemistry.

[65] Eroglu A, Toth TL, Toner M (1998). Alterations of the cytoskeleton and polyploidy induced by cryopreservation of metaphase II mouse oocytes. Fertility and sterility.

[66] Lindley EM, Jacobson JD, Corselli J, King A, Chan PJ (2001). Cryopreservation of human cumulus cells for co-cultures and assessment of DNA damage after thawing using the comet assay. Journal of assisted reproduction and genetics.

[67] Veeck, L. *Atlas of the human oocyte and early conceptus*. Vol. 2, 121–49 (Williams and Wilkins, 1991).

[68] Veeck, L. & Zaninovic, N. *An Atlas of Human Blastocysts*. 99–112 (Parthenon Publishing, 2003).

