# Supplementary material for: Galactose and its Metabolites Deteriorate Metaphase II Mouse Oocyte Quality and Subsequent Embryo Development by Disrupting the Spindle Structure
Source: Sci Rep. 2017 Mar 22;7:231. doi: 10.1038/s41598-017-00159-y (PMC5427935; doi:10.1038/s41598-017-00159-y)
Supplement: Supplementary file 1 — Supplementary Figure 1 [file 41598_2017_159_MOESM1_ESM.doc]

**Galactose and its Metabolites Deteriorate Metaphase II Mouse Oocyte Quality and Subsequent Embryo Development by Disrupting the Spindle Structure**

**Authors:** Mili Thakur^1,2^, Faten Shaeib^1^, Sana N. Khan^1^, Hamid-Reza Kohan-Ghadr^1^, Roohi Jeelani^1^, Sarah R. Aldhaheri ^1^, Bernard Gonik^1^, and Husam M. Abu-Soud^1,3^

^1^Department of Obstetrics and Gynecology, The C.S. Mott Center for Human Growth and Development, Wayne State University School of Medicine, Detroit, MI 48201, USA;

^2^Division of Genetic and Metabolic Disorders, Department of Pediatrics and Center for Molecular Medicine and Genetics, Wayne State University School of Medicine, Detroit, MI 48201, USA;

^3^Department of Biochemistry and Molecular Biology, Wayne State University School of Medicine, Detroit, MI 48201, USA.

**Corresponding Author:** Husam M. Abu-Soud, Ph.D, Department of Obstetrics and Gynecology, Wayne State University School of Medicine, The C.S. Mott Center for Human Growth and Development, 275 E. Hancock Detroit, MI 48201, Tel. 313 577-6178, Fax. 313 577-8554, E-Mail: [habusoud@med.wayne.edu](mailto:habusoud@med.wayne.edu).

**
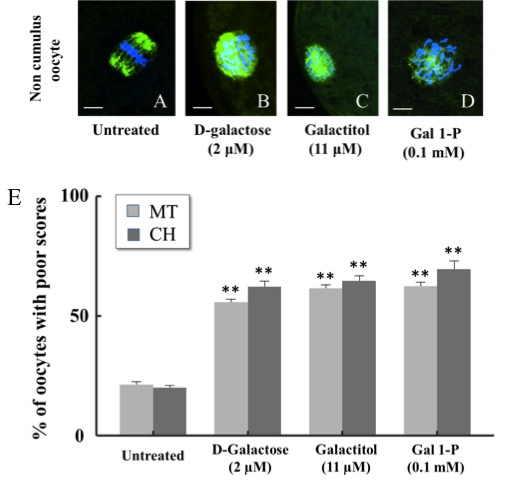
**

**Supplementary Figure 1:** **The effect of galactose and its metabolites on fresh Metaphase II mouse oocyte spindles and chromosomes.** A) Representative confocal images of metaphase II mouse spindles of fresh oocytes without cumulus cells stained with β-tubulin antibody to visualize the microtubules (MT) (green) and counterstained with DAPI to visualize chromosomes (CH) (blue). After 4 hours of incubation, various abnormal configurations of spindles were observed when oocytes were exposed to D-galactose (B), galactitol (C) or Gal 1-P (galactose 1-phosphate) (D) compared to normal spindle shapes in untreated group (A) (n=30/group). Scale bars: 1 pixel, 3 mm. Images shown are from a typical triplicated experiment. E) The percentage of oocytes with poor scores in MT structure and CH alignment in untreated oocytes compared to oocytes treated with galactose, galactitol and Gal 1-P (galactose 1-phosphate) {n=30/group}. Poor scores were significantly increased in oocytes exposed to galactose and its metabolites compared with controls in both MT and CH, indicated by ** (p<0.001 for both MT and CH). The experiment was conducted in triplicate.
